# The Mechanical and Perfusion Basis of Exercise Limitation in Apical Hypertrophic Cardiomyopathy

**DOI:** 10.1016/j.jacadv.2024.101555

**Published:** 2025-01-08

**Authors:** Rebecca K. Hughes, James Malcolmson, Ricardo P. Monteiro, Camilla Torlasco, Rhodri Davies, Luis R. Lopes, Saidi Mohiddin, Gabriella Captur, James C. Moon, Guy Lloyd

**Affiliations:** a Institute of Cardiovascular Science, University College London, London, UK; b Barts Heart Centre, Department of Cardiac Diagnostics and The Inherited Cardiovascular Diseases Unit, St Bartholomew’s Hospital, London, United Kingdom; c Centre for Advanced Imaging, William Harvey institute, Queen Mary University of London, London, United Kingdom; d Faculty of Medicine and Biomedical Science, University of Algarve, Faro, Portugal; e Department of Cardiology, IRCCS Istituto Auxologico Italiano, Milan, Italy; f Inherited Heart Muscle Conditions Clinic, Department of Cardiology, Royal Free Hospital, NHS Trust, London, United Kingdom; g MRC Unit of Lifelong Health and Ageing, University College London, London, United Kingdom

**Keywords:** apical hypertrophic cardiomyopathy, cardiac magnetic resonance imaging, cardiopulmonary exercise testing, exercise echocardiography, multimodality imaging, transthoracic echocardiography

**Figure:**
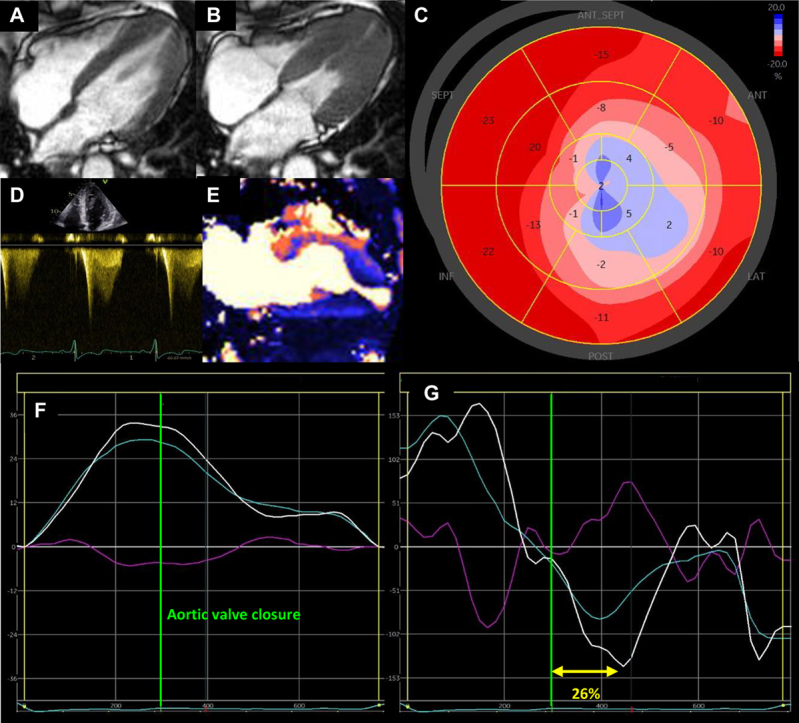

Patients with apical hypertrophic cardiomyopathy (ApHCM) commonly suffer symptoms of chest pain and dyspnea. Apical hypertrophy causes cavity obliteration early in systole, persisting into diastole; generating high pressures and creating basal to apical heterogeny in myocardial deformation across the cardiac cycle.^1^ Conventional measures of systolic function (eg, ejection fraction [EF]) may be supranormal, but other parameters may be abnormal either globally (global longitudinal strain [GLS]),^2^ or apically (longitudinal, radial and circumferential strain, including twist).^3^ Cardiac magnetic resonance (CMR) demonstrated that apical perfusion defects are a universal feature across the phenotypic spectrum.^4^ Reduced exercise capacity in HCM is widely reported; however, in ApHCM, functional limitation has been little explored, and the underpinning roles for abnormal myocardial mechanics and perfusion abnormalities are unknown. We hypothesized that patients with ApHCM would have functional limitation associated with abnormalities of global/regional myocardial mechanics (strain, twist) and myocardial blood flow (MBF).

A prospective study approved by the National Health Service Research Ethics Committee and Health Research Authority and conducted in accordance with the Declaration of Helsinki. All patients provided written, informed consent (REC 18/LO/0188 and 15/LO/0086). We recruited patients with clinically diagnosed ApHCM. Patients underwent exercise transthoracic echocardiography with breath-to-breath cardiopulmonary exercise test (CPET) (COSMED It) simultaneously (semirecumbent bicycle, ramp protocols determined individually [typically 20-W]). Early exercise was used for imaging assessment because during this phase, increased cardiac output is more reflected by augmented stroke volume rather than heart rate. We have also previously demonstrated that both EF and GLS tend to plateau at this stage. The test was terminated if one of these criteria was met (intolerable symptoms, muscular exhaustion, symptomatic hypotension, arrhythmias, significant hypertension). CMR was performed on a 1.5-T magnet using a standard clinical protocol consisting of cine imaging, stress and rest perfusion mapping with maps produced inline on the scanner using the Gadgetron framework whereby each pixel denotes MBF in ml/g/minute.

Transthoracic echocardiography postprocessing analysis used EchoPac, version 204 (GE Medical Systems) by 2 experienced readers. CMR analysis was performed by a European Association of Cardiovascular Imaging level 3 accredited cardiologist using CVI42 (Circle Cardiovascular Imaging). Statistical analysis was performed in SPSS (IBM SPSS statistic, Version 26.0) and R version 4.1.2. Continuous data were presented as mean ± SD if normally distributed, or median (IQRs) otherwise, and compared across participant groups using independent Student’s *t*-test (or paired *t*-test if within group) or Mann-Whitney *U* test, respectively. Correlation was assessed with Pearson’s or Spearman’s coefficient. A linear regression model was used to determine which exposures were associated with the outcome variable of percentage predicted peak VO_2_ (PP peak VO_2_). Unique, clinically relevant covariates with a *P* value < 0.10 were then entered into final multivariable regression models using a forward stepwise procedure and their incremental predictive value measured by the chi-square method. A 2-sided *P* value ≤0.05 was considered significant.

Forty-six patients were recruited (4 later excluded). Of the remaining 42 patients (age 54.1 ± 12 years, 81% male, body surface area 2.01 ± 0.2 m^2^), 1 did not undergo CMR (claustrophobia). 26/41 (63.4%) had apical scar. Mean maximum wall thickness was 17.5 ± 5 mm. Global and apical longitudinal strains were markedly impaired but augmented with exercise, significantly so at the apex (−11.0% [IQR: −15%, −7%] vs −12.5% [IQR: −16%,−9%], *P* = 0.201, 95% CI: 0.62-2.73 and −8.6% ± 7% vs −10.9% ± 9%, *P* = 0.011, 95% CI: 0.62-4.35, respectively). Measures of twist, twist rate, and untwist rate also augmented with exercise: LV twist (22.6° ± 9° vs 30.6° ± 7°, *P* = 0.006, 95% CI: −9.77 to −2.27), left ventricle (LV) twist rate (126.3°/s[IQR: 81,160°/s] vs 213.9°/s [IQR: 166, 236°/s], *P* < 0.001, 95% CI: −104.97 to −54.66), and LV untwist rate (−98.4°/s [IQR: −144.6,−73.9°/s] vs −177.4°/s[IQR: −249,−129°/s], *P* < 0.001, 95% CI: 51.23-114.41). This was largely determined by apical twist/twist rate as basal parameters remained unchanged. Diastolic untwisting time, which was delayed at rest (18% into diastole), became numerically more delayed (25%) during exercise, although this failed to reach significance (*P* = 0.070, 95% CI: 16.72-23.64).

GLS worsened with increased apical hypertrophy (*r* = 0.603, *P* < 0.001) and markers of impaired LV filling; lower septal (*r* = −0.421, *P* = 0.015), and lateral E’ (*r* = −0.573, *P* < 0.001). Lower apical subendocardial MBF correlated with: 1) more impaired diastolic function (lower septal S’ (*r* = −0.347, *P* = 0.041), and lateral E’ (*r* = 0.517, *P* < 0.001); 2) more impaired GLS (*r* = −0.621, *P* < 0.001); 3) apical longitudinal strain (*r* = −0.567, *P* < 0.001); and 4) longer diastolic untwisting time (*r* = −0.350, *P* = 0.039). All correlations were stronger on exercise. LVEF had no correlation with GLS (*r* = 0.139, *P* = 0.405), apical longitudinal strain (*r* = 0.015, *P* = 0.928), nor twist/untwist parameters at rest or exercise.

Peak VO_2_ was 23.4 ± 8 ml/kg/min; in 35% this was reduced (<80% predicted). The PP peak VO_2_ was worse when GLS was impaired at rest (unstandardized β coefficient [β] t = −1.583, *P* = 0.017) and exercise (β = −1.549, *P* = 0.048). It was lower with longer diastolic untwisting time on exercise (β = −0.748, *P* = 0.011) and reduced apical subendocardial MBF (β = 17.300, *P* < 0.005). On multivariate analysis, only diastolic untwisting time on exercise, when controlled for age and sex (R^2^ for model 0.469, *P* = 0.030) predicted PP peak VO_2_.

In this study, one-third of patients with ApHCM had objective evidence of functional limitation, which was associated with mechanical and microvascular abnormalities (impaired GLS and apical MBF). Patients with ApHCM had marked abnormalities of global and apical longitudinal strain, however, contrary to previous published results, our cohort demonstrated increased apical and LV twist and twist rate. Apical function was, however, still abnormal with a longer diastolic untwisting time leading to perseveration of contraction later in diastole, an effect more apparent during exercise. Functional limitation (PP peak VO_2_) was independently associated with diastolic untwisting time on exercise. These results begin to provide a narrative as to the complex interlinked structural and functional mechanisms of this disease. We hypothesize that abnormalities of myocardial contraction/relaxation lead to inefficient diastole reducing MBF and thus the oxygen supply needed for active myocardial relaxation in a feedback loop, with a net clinical effect of functional limitation.

The subendocardium is most vulnerable to ischemia and increased wall stress and predominantly affects longitudinal shortening function. Here we demonstrate a strong association between impaired apical MBF (most impaired subendocardially) and GLS, providing evidence of a link between microvascular ischemia and longitudinal contractile dysfunction (Figure 1). On a cellular level, recurrent ischemia is likely to result in myocyte death and replacement fibrosis, further attenuating GLS (and eventually overall systolic function, which is seen in the “burn-out” phase), which relates to fibrosis in HCM.^5^

**Figure 1:**
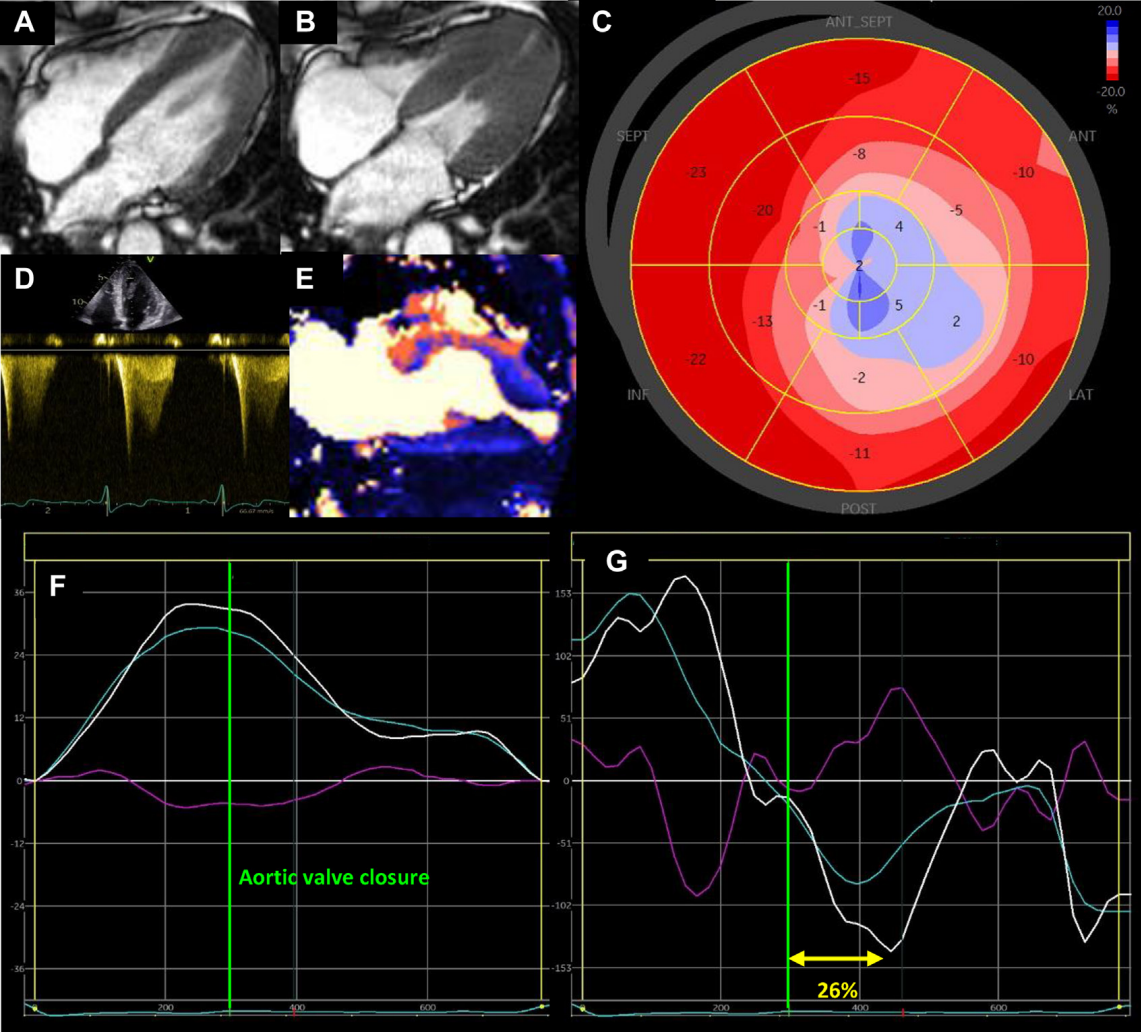
**Multiparametric Transthoracic Echocardiographic and Cardiac Magnetic Resonance Imaging Findings in a Patient With Overt Apical Hypertrophic Cardiomyopathy and an Apical Aneurysm** (A) CMR 4-chamber image in end-diastole demonstrating apical hypertrophy. (B) CMR 4-chamber image in end-systole showing apical aneurysm with cavity obliteration proximal to aneurysm. (C) Global longitudinal strain bullseye plot showing reduced and paradoxical apical strain typical of an apical aneurysm. (D) Continuous wave Doppler trace through mid-left ventricle on TTE showing classic Doppler appearance of an apical aneurysm with early systolic peak and subsequent signal dropout and a distinct paradoxical diastolic flow jet. (E) 2-chamber stress perfusion CMR map demonstrating impaired apical perfusion. (F) TTE twist graph showing increased apical and LV twist. (G) TTE twist rate graph demonstrating increased rate of apical and LV twist, but delayed peak untwist rate in early diastole at 26%. TTE = transthoracic echocardiography.

In conclusion, one-third of patients with ApHCM have functional limitation, independent of the degree of apical hypertrophy. We propose that the delay in diastolic relaxation is the unifying feature linking mechanical, functional, and physiological impairment in ApHCM by interfering with normal MBF in diastole, contributing to apical microvascular ischemia and reduced exercise capacity.

## Funding support and author disclosures

Dr Hughes is supported by the British Heart Foundation (grant number FS/17/82/33222). Dr Malcolmson is funded by a National Institute of Health Research Clinical Doctoral Research Fellowship (ICA-CDRF-2016-02-068). Dr Torlasco is supported by the Italian Ministry of Health. Dr Captur is supported by the National Institute for Health Research Rare Diseases Translational Research Collaboration (NIHR RD-TRC, #171603) and by NIHR University College London Hospitals Biomedical Research Centre. Dr Moon is directly and indirectly supported by the University College London Hospitals NIHR Biomedical Research Centre and Biomedical Research Unit at Barts Hospital, respectively. Dr Lopes is supported by an MRC UK Clinical Academic Partnership Award (CARP) MR/T005181/1. Dr Lloyd has consultancy agreement with GE and acts a speaker for GE, Phillips, Siemens, Janssen and holds research support grants from Medtronic. The remaining authors have nothing to disclose.
